# Genomic study of the response of chicken to highly pathogenic avian influenza virus

**DOI:** 10.1186/1753-6561-5-S4-S25

**Published:** 2011-06-03

**Authors:** Laura Sironi, John L Williams, Alessandra Stella, Giulietta Minozzi, Ana Moreno, Paola Ramelli, Jianlin Han, Steffen Weigend, Junxing Wan, Guerino Lombardi, Paolo Cordioli, Paola Mariani

**Affiliations:** 1 Parco Tecnologico Padano, 26900 Lodi, Italy; 2 Animal Health Department, Istituto Zooprofilattico Sperimentale della Lombardia e dell'Emilia Romagna “Bruno Ubertini”, 25124 Brescia, Italy; 3 CAAS-ILRI Joint Laboratory on Livestock and Forage Genetic Resources, Institute of Animal Science, Chinese Academy of Agricultural Sciences (CAAS), Beijing, 100193, PR China; 4 Institute of Farm Animal Genetics, Friedrich Loeffler Institut, Mariensee, D-31535, Germany; 5 DIPAV, Università degli Studi di Milano, 20133 Milano, Italy; 6 IBBA-CNR, 26900 Lodi, Italy

## Abstract

**Background:**

The host mounts an immune response to pathogens, but few data are currently available on the role of host genetics in variation in response to avian influenza (AI). The study presented here investigated the role of the host genetic background in response to *in vivo* infection with AI virus (AIV).

**Methods:**

Experimental lines of chicken and commercial crosses were experimentally infected intratracheally with 10^3^ EID_50_/bird of A/Chicken/Italy/13474/99 H7N1 highly pathogenic avian influenza virus (HPAIV). Chickens were genotyped for the *Mx* polymorphism causing the S631N mutation, and for the Major Histocompatibility Complex (MHC). Whole-genome genotyping was carried out using 60 k Single Nucleotide Polymorphism (SNP) array developed by the poultry Genome-Wide Marker-Assisted Selection Consortium (GWMASC).

**Results:**

Variability in response of different chicken lines to the HPAIV infections and some degree of resistance to AI were observed: a statistically significant effect of chicken line on the response to infection was found. There was no association between survival in healthy conditions and polymorphisms at the *Mx* gene and the MHC-*B* region. The analysis based on the 60 k SNPs provided a good clustering of the chicken lines, but no specific genetic cluster associated with response to AIV was identified.

**Conclusions:**

Neither the genotype at the *Mx* gene or MHC-*B* locus, nor for SNP spanning the whole-genome identified loci involved in variations to response to AIV infection. These results point towards the possibility that either the genetic factors affecting the response of chickens to the H7N1 HPAIV are weak, or relevant alleles were not segregating in the studied populations.

## Background

Avian influenza (AI) outbreaks are of concern as viral strains crossing the species barrier and infecting humans have been reported [e.g. 1]. The identification of genetic factors affecting resistance/susceptibility to pathogens would improve selection schemes for the development of resistant flocks of chicken.

Host response to pathogens relies on innate and adaptive immune responses. Mx proteins are antiviral molecules, which are part of the innate immune response [rev. 2], and genetic variations in the protein affect the antiviral activity. In chicken, Mx antiviral activity against vesicular stomatitis virus and H5N1 virus has been reported to be dependent on the presence of asparagine at position 631 of the molecule [3].

Products of the Major Histocompatibility Complex (MHC) play a pivotal role in both innate and adaptive immune response, and chicken MHC haplotypes have been reported to be associated with either resistance or susceptibility to infectious diseases [rev. 4]. In order to identify additional loci that are involved in variations in response to infection, the chicken genome sequence [5] and Single Nucleotide Polymorphisms (SNPs) [6] information available make possible large scale and comprehensive association studies to dissect genetic mechanisms behind the response of chickens to pathogens.

In the present work, the effect of the genetic background of a range of chicken lines on their response to *in vivo* AI virus (AIV) infection was investigated.

## Methods

### Experimental infection

In addition to those previously described [7], six additional broiler commercial lines, here referred to as lines F, G, H, I, L and M, were challenged with the A/chicken/Italy/13474/99 H7N1 highly pathogenic avian influenza virus (HPAIV). Line F was a repetition of the experimental infection of line B, but with five more birds [7]. The experimental infection was carried out on 20 9-week old birds for line F, and on groups of five adult birds each for lines G through M. The experimental protocol was as described [7]. All the experiments were conducted complying to animal care committee at the institute where this study was carried out.

### Genotyping

Genomic DNA was extracted from whole blood samples collected before the experimental infection. The 45 birds constituting lines F to M were analysed for the *Mx* gene polymorphism causing the mutation at amino acid 631 as described [7].

The total of 130 chickens (including the 85 birds previously described [7], and the 45 birds from lines F to M) were also genotyped for their variations at five microsatellite markers located within the MHC-*B* region: LEI0258 [8], MCW0371 [9,10], MCW0312 [11], and two further microsatellite loci, MHC-D (F: 5’-CTGTTGGCGTTACAGAGCT-3’; R: 5’-TTCACCCAGCAGCCTCTATC-3’) and MHC-T (F: 5’-ATGGTGGCCAAGTAAACTGGAG-3’; R: 5’- GGATCTGACAGCTGAGCGAGGT-3’). Each micosatellite marker was independently amplified. PCRs were carried out in a total volume of 12 µl, with 30 ng genomic DNA, 1.2 µl 10× buffer, 1.5 mM MgCl_2_, 0.2 mM each dNTP, 3 pmol each primer, 0.5 U *Taq* polymerase. The PCR protocol was 94°C for 3 min, 30 cycles of 94°C for 30 s, annealing temperatures 56°C (MCW0312); 60°C (MCW0371 and MHC-D); 62°C (MHC-T); 65°C (LEI0258), for 30 s, 72°C for 30 s, and a final extension step at 72°C for 7 min. The forward primer of each microsatellite marker was 5’-labelled with either FAM or HEX. PCR products were analysed on ABI PRISM 3730 DNA Analyzer (Applied Biosystems). Analysis of electropherograms and allele scoring was performed with the GeneMapper software (Applied Biosystems). Haplotypes were reconstructed using the Arlequin software, ver 3.1 [12]. Statistical analysis was carried out using four inter-dependent variables (clinical score, time course of disease, chicken line, and MHC genotype) as previously described [7], and separately for the two variables chicken lines and MHC genotypes.

The 130 chicken samples were genotyped using the 60 k SNP array developed by the poultry Genome-Wide Marker-Assisted Selection Consortium (GWMASC). Genotyping was carried out using the Illumina technology at DNA LandMarks Inc., Canada [13]. The genetic structure analysis was based on kinship of 17 chicken commercial lines. A total of 1,024 chickens were genotyped. Fourteen samples randomly chosen were genotyped twice as positive controls. GenABEL [14] was used to analyze the 60 k genotyping data within the R statistical environment [15]. The quality check on the dataset was carried out with the following thresholds: 0.95 for marker call rate, 0.95 for sample call rate, 0.0001 for minor allele frequency, as implemented in the “check.marker” function of GenABEL. Samples sharing genotype identity higher than 0.95 with another sample were excluded from the analysis. No markers were excluded following the test for Hardy-Weinberg equilibrium. Classical Multidimensional Scaling based on kinship analysis of the clean dataset was used to define samples in two-dimensions, and clusters were defined “a posteriori” based on kinship analysis.

## Results and discussion

### Clinical observations

**Figure 1:**
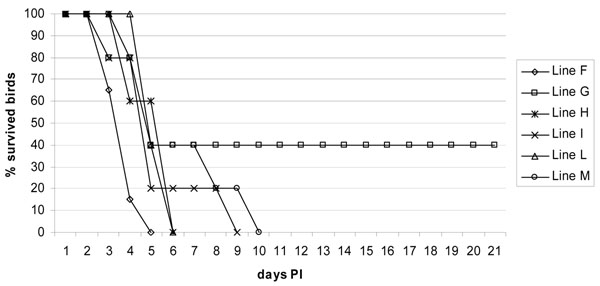
**Survival plot.** Survival curves for 6 chicken lines following experimental challenge (day "0") with HPAI H7N1 influenza virus

All birds from lines H, I, L, and M died within 6, 9, 6, and 10 days after the infections (PI), respectively (Fig. 1). Only 2 birds of line G survived the infection (the remaining 3 birds of line G died within day 5 PI). Line F was a repetition of line B [7]. All birds from the former group died within 5 days following infection.

### Genetic analyses

The six lines (F to M) were analysed for the G/A polymorphism at position 2,032 of the chicken *Mx* cDNA and were segregating for the two alleles, as previously observed for the broiler commercial lines B and C [7]. The A allele, coding for asparagine at position 631 of the protein putatively associated with higher antiviral activity [3], had frequencies ranging between 10% (lines I and M) and 90% (line G). The statistical analyses carried out on all 130 birds (including all the birds from lines A to E [7]) did not find an association between the *Mx* genotype and the response of the birds to AIV infection as previously found by Sironi and colleagues [7]. However, a significant effect of the line on clinical status after the viral challenge was found [7], thus suggesting a genetic basis to variability in response to the pathogen.

Twenty-eight MHC haplotypes reconstructed using the genotyping data across the five microsatellites were identified among the birds used in the study, constituting a total of 60 genotypes. The genotype frequency ranged from 0.77% to 11.5%, and 37 out of the 60 genotypes were unique (i.e. present only once in the population analysed). Higher homozygosity at the MHC-*B* region was observed in lines E and B compared to the other lines, confirming that these lines contained greater genetic diversity as observed for genotyping results obtained using microsatellite markers scattered through the genome (data not shown). Twelve of the 60 MHC genotypes were found among the birds that survived the infection: four birds carried unique MHC genotypes; whereas the remaining 18 surviving birds carried genotypes also present in birds that died as a result of the AIV infection. The statistical analysis, in which the effect of the two variables “line” and “MHC genotype” were estimated independently, showed that the MHC genotype did not have significant effect on the survival in healthy clinical status (*p* = 0.3574). Further association studies based on chicken groups characterised by specific serotypes are fundamental to understand if MHC plays a significant role in response and outcome of infection with AIV.

Kinship analysis of genotype data from the 60 k SNP set clustered the majority of birds into the 11 lines studied. All birds from lines A, E, I, and M clustered into separate unique clusters, whereas line C birds split into 2 clusters. However, the close proximity of the clusters suggested highly similar genetic background among these chickens. The same was observed for lines G and H, each of which split into two very closely related clusters. Additionally the clusters from these lines were in proximity indicating that they had similar genetic background. A group of birds from line C overlapped with the cluster for line D. Finally, lines B, F, and L clustered together. This was expected for lines B and F: the latter to be an experimental repetition of the former, even though chickens from lines B and F showed different responses to AIV infection. In order to further investigate a possible genetic distinction between lines B and F, an additional analysis was carried out with GenABEL using exclusively the samples from these lines. Using all permutations of possible cluster definitions (pre-setting 2, 3, 4, and 5 as potential number of clusters) individuals from the two lines were split over all available clusters, indicating that lines B and F were indeed genetically closely related.

## Conclusions

This study showed that genetic line has a statistically significant effect on the response of chickens to the H7N1 HPAIV. Nevertheless, no significant association between variability in response and polymorphisms in specific candidate loci (*Mx* and MHC-*B*) or genome-wide (60 k SNP array) was identified.

As the *Mx* gene was only genotyped for the 2,032 polymorphism, it cannot be ruled out another polymorphism, within the *Mx* gene, may influence the *in vivo* antiviral activity. It is also possible that alleles of other genes that were not segregating in the lines examined in the present study confer resistance to H7N1 HPAIV.

## List of abbreviations used

AI: avian influenza; AIV: avian influenza virus; HPAIV: highly pathogenic avian influenza virus; MHC: Major Histocompatibility Complex; SNP: Single Nucleotide Polymorphism; GWMASC: Genome-Wide Marker-Assisted Selection Consortium; PI: Post Infection.

## Competing interests

The authors declare that they have no competing interests.

## Authors' contributions

LS genotyped the 130 birds for Mx polymorphism. LS and JW genotyped the 130 birds for MHC. PR assisted in the lab work. PM and JLW conceived the main idea of the study. LS and PM drafted the paper. LS and GM performed statistical analysis under the direction of AS. AM and GL carried out the in vivo trials under the direction of PC. JH provided the MHC markers and contributed to the data analysis. SW provided two chicken lines and contributed to the data analysis. All authors contributed to the design of the study and the methods. All authors contributed to discussion of the results, revision of the paper, and approved the final manuscript.

## References

[B1] WHO | Cumulative number of confirmed human cases of avian influenza A/(H5N1) reported to WHO http://www.who.int/csr/disease/avian_influenza/country/cases_table_2010_05_06/en

[B2] Haller O Stertz S Kochs G The Mx GTPase family of interferon-induced antiviral proteins Microbes Infect 2007 9 1636 1643 10.1016/j.micinf.2007.09.010 18062906

[B3] Ko JH Jin HK Asano A Takada A Ninomiya A Kida H Hokiyama H Ohara M Tsuzuki M Nishibori M Mizutani M Watanabe T Polymorphisms and the differential antiviral activity of the chicken *Mx* gene Genome Res 2002 12 595 601 11932243 10.1101/gr.210702 PMC187515

[B4] Lamont SJ Impact of genetics on disease resistance Poult Sci 1998 77 1111 1118 9706074 10.1093/ps/77.8.1111

[B5] International Chicken Genome Sequencing Consortium Sequence and comparative analysis of the chicken genome provide unique perspectives on vertebrate evolution Nature 2004 432 695 716 10.1038/nature03154 15592404

[B6] Wong GK Liu B Wang J Zhang Y Yang X International Chicken Polymorphism Map Consortium A genetic variation map for chicken with 2.8 million single-nucleotide polymorphisms Nature 2004 432 717 722 10.1038/nature03156 15592405 PMC2263125

[B7] Sironi L Williams JL Moreno-Martin AM Ramelli P Stella A Jianlin H Weigend S Lombardi G Cordioli P Mariani P Susceptibility of different chicken lines to H7N1 highly pathogenic Avian Influenza virus and the role of *Mx* gene polymorphism coding amino acid position 631 Virology 2008 380 152 156 10.1016/j.virol.2008.07.022 18723201

[B8] McConnell SKJ Dawson DA Wardle A Burke T The isolation and mapping of 19 tetranucleotide microsatellite markers in the chicken Anim Genet 1999 30 183 189 10.1046/j.1365-2052.1999.00454.x 10442979

[B9] Buitenhuis AJ Rodenburg TB Van Hierden YM Siwek M Cornelissen SJ Nieuwland MG Crooijmans RP Groenen MA Koene P Korte SM Bovenhuis H van der Poel JJ Mapping quantitative trait loci affecting feather pecking behavior and stress response in laying hens Poult Sci 2003 82 1215 1222 12943291 10.1093/ps/82.8.1215

[B10] Fulton JE Juul-Madsen HR Ashwell CM Mccarron AM Arthur JA O'Sullivan NP Taylor RL Jr Molecular genotype identification of the *Gallus gallus* major histocompatibility complex Immunogenetics 2006 58 407 421 10.1007/s00251-006-0119-0 16738938

[B11] Crooijmans RPMA Dijkhof RJM van der Poel JJ Groenen MAM New microsatellite markers in chicken optimized for automated fluorescent genotyping Anim Genet 1997 28 427 437 10.1111/j.1365-2052.1997.00205.x 9589584

[B12] Excoffier L Laval G Schneider S Arlequin (version 3.0): an integrated software package for population genetics data analysis Evol Bioinform Online 2005 1 47 50 19325852 PMC2658868

[B13] DNA Landmarks http://www.dnalandmarks.ca

[B14] Aulchenko YS Ripke S Isaacs A van Duijn CM GenABEL: an R library for genome-wide association analysis Bioinformatics 2007 23 1294 1296 10.1093/bioinformatics/btm108 17384015

[B15] The R project for statistical computing http://www.r-project.org

